# Silkworm Gut Fibres from Silk Glands of *Samia cynthia ricini*—Potential Use as a Scaffold in Tissue Engineering

**DOI:** 10.3390/ijms23073888

**Published:** 2022-03-31

**Authors:** Salvador D. Aznar-Cervantes, Ana Pagán, María J. Candel, José Pérez-Rigueiro, José L. Cenis

**Affiliations:** 1 Departamento de Biotecnología, Genómica y Mejora Vegetal, Instituto Murciano de Investigación y Desarrollo Agrario y Ambiental (IMIDA), La Alberca, 30150 Murcia, Spain; sdac1@um.es (S.D.A.-C.); mjcandelc@gmail.com (M.J.C.); josel.cenis@carm.es (J.L.C.); 2 Centro de Tecnología Biomédica, Universidad Politécnica de Madrid, Pozuelo de Alarcón, 28223 Madrid, Spain; jose.perez@ctb.upm.es; 3 Departamento de Ciencia de Materiales, ETSI Caminos, Canales y Puertos, Universidad Politécnica de Madrid, 28040 Madrid, Spain; 4 Biomedical Research Networking Center in Bioengineering, Biomaterials and Nanomedicine (CIBER-BBN), 28223 Madrid, Spain

**Keywords:** silk fibroin, *Samia cynthia ricini*, *Bombyx mori*, silkworm gut fibre, tissue engineering

## Abstract

High-performance fibroin fibres are ideal candidates for the manufacture of scaffolds with applications in tissue engineering due to the excellent mechanical properties and optimal biocompatibility of this protein. In this work, the manufacture of high-strength fibres made from the silk glands of *Samia cynthia ricini* is explored. The glands were subjected to soaking in aqueous dissolutions of acetic acid and stretched to manufacture the fibres. The materials produced were widely characterized, in terms of morphology, mechanical properties, crystallinity and content of secondary structures, comparing them with those produced by the standard procedure published for *Bombyx mori*. In addition, mechanical properties and biocompatibility of a braided scaffold produced from these fibres was evaluated. The results obtained show that the fibres from *B. mori* present a higher degree of crystallinity than those from *S. c. ricini*, which is reflected in higher values of elastic modulus and lower values of strain at break. Moreover, a decrease in the elongation values of the fibres from *S. c. ricini* was observed as the concentration of acetic acid was increased during the manufacture. On the other hand, the study of the braided scaffolds showed higher values of tensile strength and strain at break in the case of *S. c. ricini* materials and similar values of elastic modulus, compared to those of *B. mori*, displaying both scaffolds optimal biocompatibility using a fibroblast cell line.

## 1. Introduction

The research on biomaterials derived from silk fibroin (SF) has experienced an exponential growth over the last three decades due to its excellent mechanical properties and optimal biocompatibility, in addition to the great versatility of presentations in which it can be processed, such as gels, sponges, fibres, films, nanoparticles or tubes [1,2,3,4,5,6,7,8]. Most scientific papers and reviews on fibroin applications in the biomedical field have focused on the SF produced by the mulberry silkworm, *Bombyx mori* [9]. However, there are many silk-producing arthropods with interesting properties to explore, including *Samia cynthia ricini*, also known as eri silkworm. Eri SF presents a primary structure composed of 100 repeats of alternating poly-(L)-alanine (PA) and glycine domains, while *B. mori* SF is mainly composed of glycine, alanine and serine residues [10], with a higher content of glycine than in the case of fibroin from *S. c. ricini* [11]. These differences in the biochemical composition of both fibroins more than likely have repercussions on the mechanical properties of raw silks and the SF biomaterials derived from them, as well as on their functionality, and hence the interest in exploring them. Fibroin from *S. c. ricini* has previously been used in some scientific works to make scaffolds, extracting it directly from the silk glands of mature worms [11] or dissolving it in solvents such as trifluoroacetic acid [12,13,14], ionic liquids [15] or calcium nitrate tetrahydrate [16]. However, its use in this sense is much less explored than in the case of *B. mori*, despite the fact that there are some indications of potential advantages in terms of cell adhesion and proliferation on fibroin films produced from eri silkworms compared to those using fibroin from mulberry silkworms *(B. mori*) [16], as well as in comparisons with electrospun materials from tasar fibroin (produced by the worm *Antheraea mylitta*) and *B. mori*. [12].

Biomimetic approaches to SF processing are gaining the attention of the scientific community in order to produce fibres with optimal mechanical properties that match or exceed those of natural silk fibres. For this reason, different processes emulating what happens during the natural spinning of fibroin should be investigated; for example, the extrusion of fibroin dissolutions through small diameter tubular structures, similar to the reduction in diameter of the silk duct of the glands as it approaches the spinneret, as well as the processes that concern the acidification along the silk gland, or even the shear forces or stretching processes that the worm itself exerts with its head movement when making the cocoon [17]. In this sense, quite successful methods have been achieved using solvents such as hexafluoroisopropanol (HFIP) and controlled extrusion processes [18] or through mild coagulating chemistries with the technique called straining flow spinning [19,20]. In this context, the manufacture of silkworm gut fibres (SGFs) arises as an alternative procedure to natural spinning, based on an ancient method of production of fishing lines and sutures, which constituted an important industry in the southeast coast of Spain, especially in the Region of Murcia [21]. Its manufacturing procedure, known since the 18th century, consisted of soaking the silk glands of *B. mori* in an acetic acid dissolution and stretching them by hand, leading to the so-called SGFs (traditionally known as “hijuelas” in Spanish and “crins de Florence” in French). This activity went into decline from the first half of the 20th century, coinciding with the use of nylon and other synthetic fibres for these purposes [21,22]. The recent rediscovery of this type of material, as well as the optimization of its manufacture under controlled laboratory conditions, have allowed a detailed characterization of its microstructure and mechanical behaviour, also entailing a deep understanding of the spinning process [23,24]. The excellent mechanical properties of these high-performance fibres, that exceed the strain at break values of the natural material (degummed silk) and present values of work to fracture comparable to native silkworm silk [23], as well as the ideal biocompatibility of SGFs, have led our research group to explore their potential uses as scaffolds for tendinous or ligamentous tissue engineering [25] or as implantable and biocompatible light-diffusing fibres for the stimulation of cell proliferation using red light [26]. On the other hand, it has also been possible to produce this type of fibres using spider silk glands from *Nephila clavipes* [27] and *Nephila inaurata* [28], following a procedure similar to that described for *B. mori*. But, as far as we know, there is no scientific work that has investigated its production from the glands of *S. c. ricini*.

Pathologies of tendons and ligaments, as well as the problems that their rupture or damage entails, make research on new resorbable biomaterials necessary, so that they can be implanted and temporarily replace the damaged tissue until its complete regeneration. Nowadays, the use of autografts, allografts, xenografts or replacement prosthesis comprise the most common treatments in these clinical conditions [29], but all of them can lead to problems such as complicated surgeries [30], morbidity of patients with involvement of the donor area or even the induction of immune responses or the transmission of diseases. Lastly, artificial implants often fail in the long term [31].

Other approaches to address this type of clinical conditions explore the use of natural biomaterials such as collagen [32,33,34,35,36,37] or synthetic polymers as scaffolds. As examples, we can mention the use of polylactide-co-glycolide acid (PLGA) [38], poly-ε-caprolactone (PCL) and poly-L-lactide acid (PLLA) [39], as well as many other combinations of different polymers [40,41]. But the mechanical properties of these materials are still far from meeting the requirements of the native tissues. Due to its high strength, elasticity and toughness, several works have previously stated the potential use of fibroin in order to produce scaffolds with application in tendinous or ligamentous tissue engineering. This has been achieved by means different approaches, such as a knitted silk scaffolds combined with a collagen matrix [35], silk fibre wire-rope scaffolds [42,43], ectopic tissue engineered ligaments with fibroin and collagen into their composition [44], gelatine/fibroin hybrid scaffolds [45], photoactivated nanofiber graft materials for augmented Achilles tendon repair [46] or even triphasic silk-based grafts for functional regeneration of ligament-bone interface [47].

Given the above and knowing the interesting properties of SGFs, this work proposes the manufacture of this type of fibres from the silkworm *S. c. ricini* using different treatments, as well as comparing them with those produced from *B. mori* using the standard procedure developed by our research team. Therefore, relevant information is provided regarding its physicochemical characterization and its potential use in tissue engineering, evaluating for this purpose braided scaffolds made from both eri and mulberry silkworm gut fibres (SGFB).

## 2. Results and Discussion

The most relevant results after an extensive characterization of the SGFs obtained from the eri silkworm are presented below. These fibres will be referred to hereinafter as Scr SGF Ac 2%, Scr SGF Ac 10% or Scr SGF Ac 20%, depending on the acetic acid solution used during their manufacture. Interesting data on its topography, crystallinity, mechanical behaviour and biocompatibility are provided, comparing them with the fibres obtained from the mulberry silkworm. These fibres produced from *B. mori* will hereinafter be referred to as Bm SGF Ac 2%.

### 2.1. Optical Microscopy

**Figure 1:**
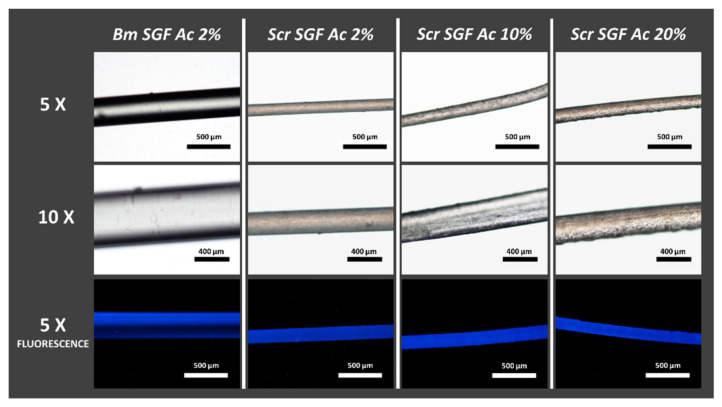
Micrographs of SGFs produced from silk glands of *S. c. ricini* (Scr SGF) under different experimental conditions and from *B. mori* (Bm SGF). The abbreviations Scr SGF Ac 2%, Scr SGF Ac 10% and Scr SGF Ac 20% refer to the fibres obtained from eri silkworms, using acetic acid dissolutions at 2%, 10% and 20% *v*/*v*, respectively, while those obtained from mulberry silkworm are named as Bm SGF Ac 2%. The images were acquired at different magnifications; 5× (scale bar 500 µm) and 10× (scale bar 200 µm).

As can be seen in Figure 1, all the manufacturing treatments of the Scr SGFs with different concentrations of acetic acid in aqueous dissolution were successful in terms of their feasibility. Their diameters were similar, but smaller than those of the Bm SGFs, obtained from *B. mori* silk glands.

The precise data on the diameters of these fibres, along with their mechanical characterization, are widely described later. All the materials produced showed a strong autofluorescence, an intrinsic property of silk fibroin protein from *B. mori* [48], when observed under a microscope using a DAPI filter and an ultraviolet light source. Consequently and as expected, Bm SGFs also showed this characteristic autofluorescence, together with Scr SGFs.

Likewise, in Figure 1 it can be seen that as the concentration of acetic acid dissolution increases in the manufacturing process, the homogeneity of the surface of the fibres apparently decreases. The surface of the fibres made with 2% *v*/*v* acetic acid dissolution seems to be smoother than in the other treatments.

### 2.2. Crystallinity Index

**Figure 2:**
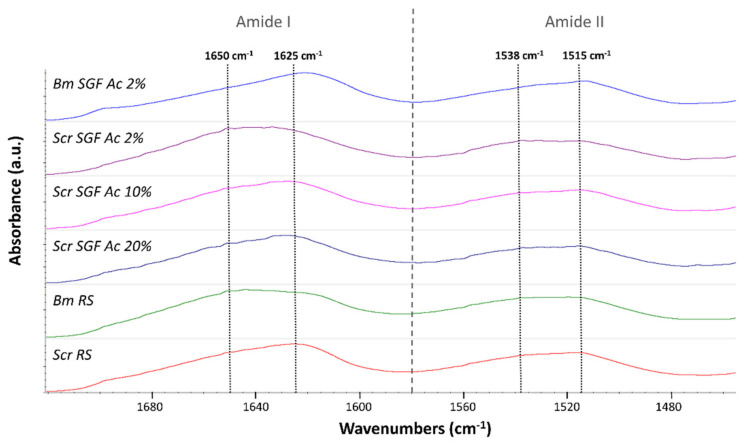
Infrared spectra of silkworm gut fibres produced from silk glands of *S.c. ricini* (Scr SGF) under different experimental conditions and from *B. mori* (Bm SGF), as well as those correspondent to raw silk fibroins produced by both species of silkworms (named as Scr RS and Bm RS, respectively). The figure focuses on amide peaks I and II, highlighting the wavenumbers whose absorbances have been used to calculate the crystallinity indices, 1625 and 1650 cm^−1^ (for the amide I peak) and 1515 and 1538 cm^−1^ (for the amide II peak).

It is important to investigate the molecular conformation of the component proteins of a specific material in order to understand to a great extent its physical properties. As explained in Section 3, by calculating crystallinity indices, based on the infrared spectra of fibroin, the molecular structure and crystallinity of this protein can be explored in a simple way. This is performed by analysing the prevalence of the signals characteristic of β-sheet structures (crystalline) over random coil structures (amorphous). Some authors have focused their works on calculating simple ratios between the characteristic intensities of peaks correspondent to these two structures, by analysing the amide III peak [49,50], or the amide I and II peaks [51], and some of them expressing it as a percentage [52,53]. The calculation used in this work (Table 1) is founded on that proposed by Jao et al. [51], based on a simple ratio between values of absorbance of β-sheets and random coils in the amide I and II peaks. As random coils show strong peaks at 1650–1660 cm^−1^ (amide I) and 1535–1545 cm^−1^ (amide II), and β-sheets show peaks at 1625–1640 cm^−1^ (amide I) and 1515–1525 cm^−1^ (amide II) [51,54], the values of crystallinity indices were calculated as the ratio between the measured absorbance at 1625 and 1650 cm^−1^ (for the amide I peak) and between 1515 and 1538 cm^−1^ (for the amide II peak) (Figure 2).

Table 1 summarizes the data obtained for the crystallinity indices of the different treatments. In addition to the manufactured fibres, the crystallinity indices of the raw silk fibroins (RS) corresponding to both species were evaluated for comparative purposes, the ones produced by *B. mori* or *S. c. ricini* abbreviated as Bm RS or Scr RS, respectively.

Regarding the statistical analysis, no significant differences were detected in the crystallinity index of the amide II peak in any of the treatments (ANOVA, *p* > 0.05). On the contrary, the crystallinity index of the amide I peak showed significant differences (ANOVA, *p* < 0.05), and the fibres produced from *B. mori* (Bm SGF Ac 2%) presented statistically higher values than the rest of the treatments (Bonferroni, *p* < 0.05). In addition, RS of *S. c. ricini* was more crystalline, compared to that of *B. mori* (Bonferroni, *p* < 0.05).

No statistical differences were detected between the different manufacturing treatments of Scr SGFs (Bonferroni, *p* > 0.05), despite the fact that the average values of the crystallinity indices augmented as the concentration of acetic acid used in their manufacture increased.

The values obtained in this study are in the range of those proposed by other authors for electrospun fibroin biomaterials [55].

### 2.3. Microstructural Characterization

The microstructural characterization of the fibroin, present in the materials studied, was carried out by deconvolution of the amide I peak (1720–1580 cm^−1^), founded on what was established by other authors [20]. Vibrational band assignments were based on the data summarized in Table 2. This analysis allowed us to confirm the trends observed in the calculation of the crystallinity indices, as well as to know more precisely the content on secondary structures of this protein.

The data presented in Table 3 show the results regarding the β-sheet, α-helix and random coil content, as they are the most relevant and clarifying structures to understand the differences between the materials studied. The results obtained are in the range of those stated by other authors using different methods for the deconvolution of the amide I peak in relation to fibroin fibres from *B. mori* [19,57,58], annealed fibroin films [5] and porous scaffolds of fibroin from *S. c. ricini* [15], among others. As examples, we can mention the average values stated by Madurga et al. for degummed SF in terms of secondary structures, around 46%, 12% and 16% for β-sheet, α-helix and random coil content, respectively [19], or the value of 48.7% for β-sheet content reported in crosslinked eri fibroin scaffolds by Silva et al. [15].

The statistical analysis revealed differences in β-sheet content (ANOVA, *p* < 0.05). The SGFs produced from *B. mori* presented a significantly higher content of these crystalline structures (59.2%) compared to those produced from *S. c. ricini* (≈49%), as well as superior to that of its own RS (44.7%) (Bonferroni, *p* < 0.05), and statistically equivalent to that of the RS of *S. c. ricini* (Bonferroni, *p* > 0.05).

Regarding the content of α-helices, the calculated values of this structure were not very illustrative. A clearly lower percentage was obtained in the case of Bm SGF Ac 2% (8.5%) than in the rest of the treatments, but the differences were only significant (Bonferroni, *p* < 0.05) in the comparison with the treatment Scr SGF Ac 20% (12%) and with Bm RS (11.6%), coinciding with the highest mean values of all those studied.

Much more clarifying were the data related to the content in random coil, a secondary structure that indicates a disorganized or amorphous protein state. In this sense, the material with the lowest content was the SGF produced from *B. mori* (11.3%), confirming the higher degree of molecular organization that denoted its greater content in β-sheet and the upper value of the crystallinity index, both previously exposed. This value was significantly lower than that of all the treatments studied (Bonferroni, *p* < 0.05), which were statistically equivalent among them (Bonferroni, *p* > 0.05), and ranged between 17% and 20%. As it will be explained later, the higher degree of crystallinity of the Bm SGFs, compared to those produced from the eri silkworm (Scr SGFs), entails implications in the mechanical behaviour of the fibres in a dry state.

It is important to highlight the fact that the content of all the studied secondary structures, in the case of *B. mori*, is different between its RS and the Bm SGFs produced (Bonferroni, *p* < 0.05). However, in the case of RS from *S. c. ricini* and its Scr SGFs, these values were detected as statistically equivalent in all treatments studied (Bonferroni, *p* > 0.05).

### 2.4. Mechanical Properties

**Figure 3:**
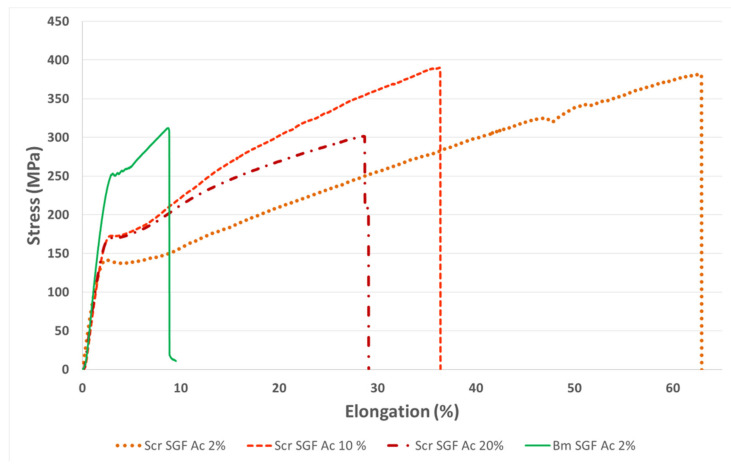
Stress–strain curves obtained during tensile tests of SGFs from silk glands of *S. c. ricini* (Scr SGF) and from *B. mori* (Bm SGF). The abbreviations include at the end the concentration of acetic acid dissolution used during its production (2%, 10% or 20% *v*/*v*).

The mechanical characterization of individual fibres, in the dry state, was carried out in order to visualize differences between them, either due to the species used or to the manufacturing procedure (concentration of acetic acid dissolution). Thus, being able to correlate the aspects related to the molecular structure with the mechanical properties obtained and therefore address a broad view of this novel material. The values of the tensile strength (MPa), strain at break (%) and elastic modulus (GPa) were calculated from the stress–strain curves recorded during the tensile tests (Figure 3), and are summarized in Table 4.

The average values of tensile strength were detected as equivalent during the statistical analysis in all the SGFs produced (ANOVA, *p* > 0.05). However, it is noteworthy that the treatment that reached the highest mean value was that of SGFs obtained from *S. c. ricini* using the acetic acid dissolution at 2% *v*/*v* (388 MPa), also displaying a much lower standard deviation, and therefore being more reproducible in terms of tensile strength. This protocol has also been considered optimal in the production of SGFs from *B. mori*, as has been pointed out in previous works [23,25,26]. These values of tensile strength are in the same range of those previously described in spider silk guts produced from *Nephila inaurata* [28] or even exceed the maximum values obtained in *Nephila clavipes* tubuliform silk guts (376 MPa) [27], both performed in a similar way.

The values of strain at break were significantly different in all the comparisons carried out (Bonferroni, *p* < 0.05), decreasing in the case of SGFs from *S. c. ricini*, as the concentration of acetic acid dissolution used for its manufacture increases. This is maybe due to the imperfections and roughness that were generated during this process, probably because it is harsher at concentrations higher than 2% *v*/*v*, and changes in the molecular conformation of fibroin occur faster and in a more disorganized way. However, this is only a hypothesis based on the presence of this stated roughness on the fibres and should be investigated more deeply in future works. The lowest value of strain at break was that of the SGFs from *B. mori* (7.9%). This fact is directly correlated with the higher content in β-sheet, higher crystallinity index and lower content in random coil, when compared with the others fibres, as previously explained. The highest average value of strain at break was that of Scr SGF Ac 2% (65.0%), which greatly exceeds that of all the SGFs previously described in the scientific literature, both those made using silk glands from mulberry silkworms [23] and those made from spiders [24,27,28]. The great values of strain at break in Scr SGFs can be explained as a consequence of the characteristic sequence of poly-L-alanine (PA) and glycine-rich motifs in fibroin from *S. c. ricini*. The latter are basically in the random coil state and provide elasticity to the fibre produced by eri worms, while PA blocks tend to adopt a β-sheet-like conformation [59]. This is different from the fibroin of *B. mori*, which basically consists of a (GAGAGS)n repeat sequence. Moseti et al. [60] stated also that the larvae of *S. c. ricini* keep some α-helices untransformed to impart flexibility in the native silk. Curiously, that same flexible behaviour is observed in the Scr SGFs analysed in this work. This statement makes sense if we observe the great similarity in content of secondary structures between the RS of *S. c. ricini* and its SGFs.

The statistical analysis also detected differences in the values of elastic modulus (ANOVA, *p* < 0.05). The mean value of SGFs produced from *B. mori* (11.6 GPa) was significantly higher than those from *S. c. ricini* manufactured with 2% (8.7 GPa) and 10% *v*/*v* (8.7 GPa) acetic acid dissolutions (Bonferroni, *p* < 0.05), and statistically equivalent to those manufactured with acetic acid at 20% *v*/*v* (9 MPa). In the latter case, the difference was probably not detected because it presented a higher standard deviation (Bonferroni, *p* > 0.05). This greater value of elastic modulus in Bm SGF Ac 2% is correlated with the higher degree of crystallinity as observed in the infrared spectroscopy analysis, performed also in the dry state.

The average diameters of the fibres produced from *S. c. ricini* were statistically equivalent (Bonferroni, *p* > 0.05), ranging between 133 and 156 µm. The fibres produced from *B. mori* presented a significantly larger diameter (248 µm) than those from *S. c. ricini* (Bonferroni, *p* < 0.05) which could be attributed to anatomic differences in the silk glands of each species.

On the other hand, the mechanical properties of braided scaffolds in the wet state were evaluated (Table 5), in order to analyse their behaviour in an environment similar to in vivo conditions. For this purpose, they were hydrated in PBS 1× for at least 48 h before performing the tensile tests. The scaffolds produced with SGFs from *B. mori* (Bm SGFB) were compared with others made with SGFs from *S. c. ricini* (Scr SGFB), using acetic acid dissolution at 2% *v*/*v* in both cases, since these fibres presented optimal mechanical properties and a more homogeneous surface and its manufacturing method is identical to that of the standard SGFs from mulberry silkworm.

**Figure 4:**
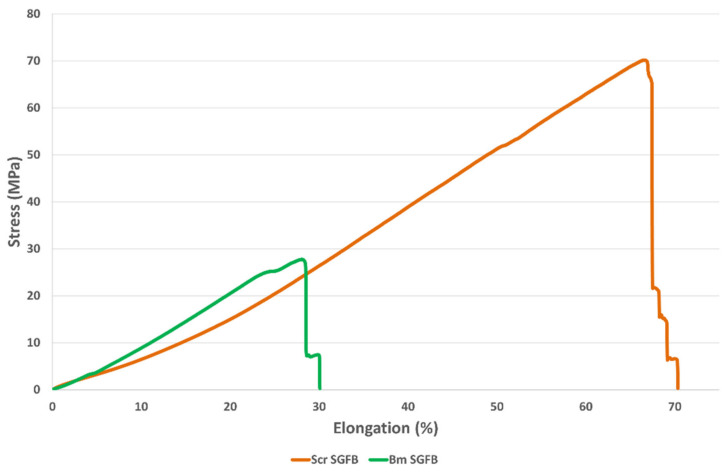
Stress–strain curves obtained during tensile tests of braided scaffolds manufactured from silk glands of *S. c. ricini* (Scr SGFB) and from *B. mori* (Bm SGFB).

The braided scaffolds of *S. c. ricini* showed higher values of tensile strength and strain at break (65.0 MPa and 66.3%) than those of *B. mori* (35.6 MPa and 34.4%) (ANOVA, *p* < 0.05) (Figure 4). These values could be adequate to address in vivo studies using this type of biomaterials, since ultimate tendon stress values in the range of 50 to 100 MPa are normally reported [61], and similar values can also be found in the scientific literature for ligaments such as sheep ACL (53.6 MPa) [43]. On the other hand, the values of strain at break of both studied scaffolds were similar to the one proposed by Altman et al. (33%) for a human anterior cruciate ligament (ACL) [42].

The values of elastic modulus were statistically equal for both scaffolds (ANOVA, *p* > 0.05). This result is interesting because it means a change in what was observed, regarding the comparison of the individual SGFs in the dry state. These presented a higher value of elastic modulus in the case of SGFs from *B. mori* when compared with those from *S. c. ricini*, as previously explained, and this fact denotes the importance of the degree of hydration in the mechanical performance of this type of fibroin materials. The average values of elastic modulus (around 100 MPa) could be suitable to research on tissue engineering of various tendons and ligaments, as they are in the range of the one stated for internal lateral ligament (61 MPa), posterior cruciate ligament (67 MPa), ACL (64 MPa) or patellar tendon (100 MPa) [62,63,64,65], as examples.

Our results improve some of those previously stated in biomaterials for tendon and ligament tissue engineering containing fibroin in their composition. For example, strain at break values of 15% are described in biphasic SF scaffolds, presenting an elastic modulus ranging from 0.7 to 1.3 MPa [66]. In this sense, Ran et al., reported higher values of elastic modulus (49.49 MPa) for ectopic tissue-engineered ligaments manufactured with silk and collagen [44]. Other authors obtained values of tensile strength and elastic modulus of 13.5 MPa and 21.7 MPa, respectively, in collagen/polyurethane/silk scaffolds for tendon tissue engineering [67]. On the other hand, Altman et al. stated strain at break values of 38.6% in their work using six cord wire-rope matrices for ACL tissue engineering [42]. This value is in the same range as that obtained by us in the braided scaffolds produced from glands of *B. mori*, but is exceeded by those of *S. c. ricini*.

Finally, the cross-sectional area of both scaffolds, calculated by measuring their thickness and width, was statistically lower in the case of Scr SGFB (ANOVA, *p* < 0.05). This result is not very relevant, since the dimensions of these can be varied by braiding more or fewer fibres to adapt them to the desired hypothetical in vivo study model, being able to adapt, in the same way, the values of maximum force that these resist, and mimicking those of the tissue to be replaced, as previously stated by our research group, in a study using braids of SGFs from *B. mori* [25]. 

### 2.5. Biocompatibility and Proliferation Study

**Figure 5:**
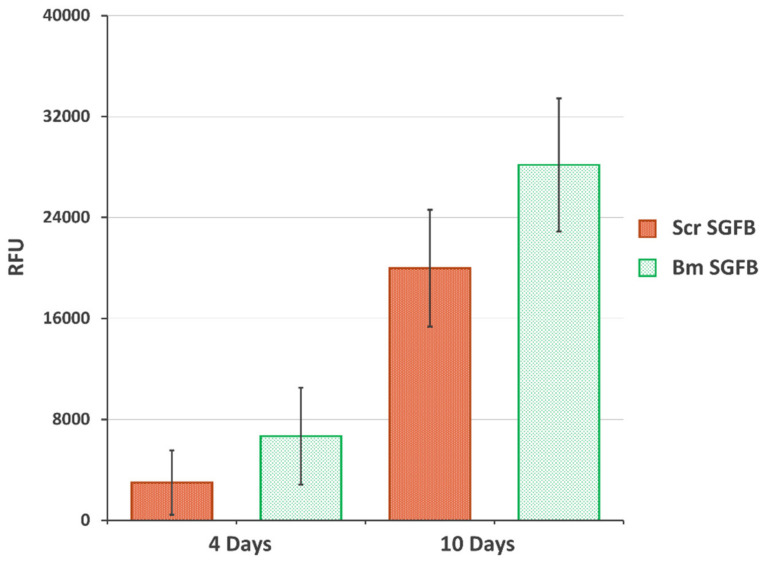
Proliferation of the human dermal fibroblasts growing on the silkworm gut fibre braids produced from silk glands of *S. c. ricini* (Scr SGFB) and from *B. mori* (Bm SGFB) at 4 days and 10 days after seeding. Data are expressed as the average values of relative fluorescence units (RFU) (570–610 nm) ± SD (*n* = 4) of the PrestoBlue assay.

The final aim of this work was to evaluate the potential use of SGFs from *S. c. ricini* for tissue engineering applications, as other authors have settled with dissolved silk or SF directly extracted from silk glands from eri silkworms. For this purpose, we evaluated the cellular adhesion and proliferation of human dermal fibroblasts on the SGFBs as crucial factors for integrating the scaffold into a biological system. The cellular viability and proliferation of cells growing onto Scr SGFB were progressive and in similar manner to those growing onto Bm SGFB, with no statistical differences observed at the different experimental times (4 and 10 days after cell seeding) (Figure 5). These results are in accordance with previous scientific works that stated the good biocompatibility of fibroin from *S. c. ricini* in different formats of scaffolding for tissue repair [11,12,13,14,15,16], which justifies and motivates further research on the potential use of SGFs produced from eri silkworm in the field of regenerative medicine.

**Figure 6:**
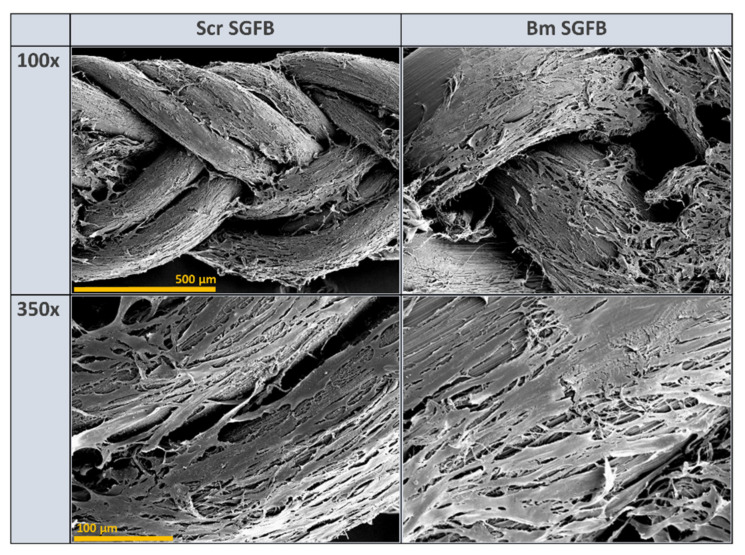
Scanning electron microscopy micrographs of human dermal fibroblasts growing on the silkworm gut fibre braids produced from silk glands of *S. c. ricini* (Scr SGFB) and from *B. mori* (Bm SGFB) at 10 days after seeding and at different magnifications: 100× (scale bar 500 µm) and 350× (scale bar 100 µm).

Biocompatibility is one of the most commonly used terms to describe appropriate biological requirements of a biomaterial to be used as a medical device. This statement of biocompatibility is extensively based in a no-cytotoxic response accompanied by normal cell growth, with a good cellular spread and viability that causes no adverse host response, and it is considered as one of the most relevant properties of silk fibroin in biomedicine applications [1,4,68]. In concordance with this assertion, the visualization of the studied scaffolds by scanning electron microscopy (SEM) confirmed the optimal and homogeneous cell attachment and spreading along with the experiment, as well as no changes or alterations in the normal morphology of these cells, with a high percentage of the scaffold being colonized by fibroblasts at 10 days after seeding in both silkworm gut fibre braids produced from silk glands of *S. c. ricini* and from *B. mori* (Figure 6).

Given the above results, it can be inferred that the SGFBs produced from *S. c. ricini* worms have great potential in the field of tissue engineering.

## 3. Materials and Methods

### 3.1. Silkworm Rearing and Preparation of SGFs

*S. c. ricini* silkworms were reared with *Ricinus communis* leaves in the facilities of IMIDA (Murcia, Spain) during 35 days at 22 ± 1 °C and 40–50% RH, until reaching its maximum weight (around 7 g per specimen), immediately prior to spinning the cocoons. *B. mori* silkworms (Italian poly-hybrid [79 × 719] × [126 × 125]), kindly supplied by Dr. S. Cappellozza (CRA-API), were reared with mulberry leaves in similar conditions, until the fifth instar (also before starting the spinning process).

**Figure 7:**
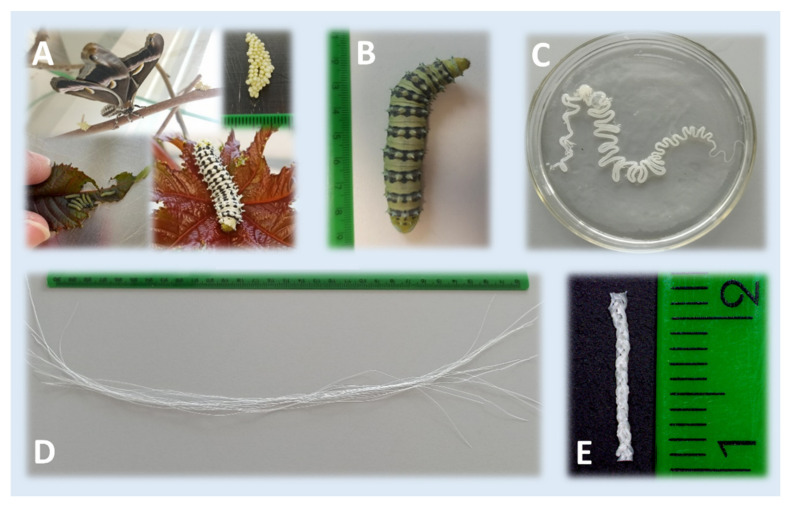
Illustrative images of the rearing of *S. c. ricini* at different stages of development (**A**), the appearance of the mature worm (**B**), the silk gland in acetic acid (**C**), a macroscopic view of the fibres produced after stretching the silk glands and cutting them into pieces (**D**) and the scaffold derived from braiding silkworm gut fibres (**E**).

The preparation of the *S. c. ricini* SGFs was carried out following a protocol similar to that described by our research group for *B. mori* SGFs in previous works [23,25,26]. Briefly, fifth instar larvae were anesthetized by exposure to a temperature of 4–5 °C for 15 min prior to sacrifice. The sericigen glands were extracted by dissecting the caterpillars dorsally along their body. Then, they were washed in distilled water and immersed in aqueous acetic acid dissolutions at 2%, 10% or 20% *v*/*v* for 2 min. After that, each gland was manually stretched, leading to the formation of a translucent fibre (sometimes exceeding 3 m in length). The resultant fibres were subsequently cut into pieces of 30 cm in length. Figure 7 illustrates the rearing of *S. c. ricini*, as well as the appearance of the mature larvae, the silk gland in acetic acid and some fibres produced after stretching the silk glands.

In the same way, a batch of SGFs was produced from *B. mori* silk glands, using only 2% *v*/*v* acetic acid dissolutions (optimal concentration established in a previous work for this species [23]), in order to compare the manufacturing treatments used employing *S. c. ricini* for the first time in this study.

### 3.2. Optical Microscopy

In order to characterize the topography and appearance of the fibres produced, they were visualized, after drying, using an optical microscope (Nikon Eclipse 50i, Tokio, Japan) at different magnifications (5×, 10× and 20×). In addition to bright field observation, fluorescence observation was carried out using a DAPI-1160A filter (excitation: 377–398 nm; emission: 417–477 nm). At least 3 fibres were observed per treatment.

### 3.3. Attenuated Total Reflectance Fourier Transformed Infrared Spectroscopy (ATR-FTIR)

ATR-FTIR was employed in order to analyse the potential structural differences of the different SGFs fabricated and those corresponding to the RS produced by both species. Each spectrum was acquired on a Nicolet iS5 spectrometer, equipped with an iD5 ATR accessory (Thermo Scientific, Waltham, MA, USA) controlled with the Omnic software (Ver. 9.3.30), measuring in absorbance mode with a resolution of 4 cm^−1^, a spectral range of 4000–550 cm^−1^ and 64 scans.

#### 3.3.1. Crystallinity Index Calculation

The change in the conformation of fibroin materials can be evaluated calculating the crystallinity index as the ratio of the absorbance of β-sheet to the absorbance of random coil for each amide band [51]. This measurement has been used in previous works as a simple approximation to comparatively evaluate the degree of crystallinity of fibroin materials produced from different species of Lepidoptera [49,50,51,53]. In this sense, infrared crystallinity indices of amide I and amide II peaks of fibroin were calculated for all the treatments as the ratio between the measured absorbance at 1625 and 1650 cm^−1^ (for the amide I peak) and between 1515 and 1538 cm^−1^ (for the amide II peak), as previously stated by other authors [51].

#### 3.3.2. Microstructural Characterization

Microstructural characterizations were carried out in order to investigate more deeply the content on secondary structures of the fibroin, focusing on the amide I peak, and based in the work by Madurga et al. [20]. Briefly, the amide I peak (1720–1580 cm^−1^) was decomposed into 9 Gaussian functions (full width at half height: 8 cm^−1^), and each one was assigned to a secondary structure following the work by Jung, as resumed in Table 2 [56]. The relative percentage of the secondary structures was calculated from the areas of the corresponding Gaussian functions. The entire fitting process was performed with the Omnic 9 software.

The data on the content of β-sheet, α-helix and random coil secondary structures are presented in Section 2, as they are the most relevant and clarifying to understand the differences between the different materials studied.

All determinations related to infrared spectroscopy were carried out on SGFs produced from silk glands of *S. c. ricini* (Scr SGF) under different experimental conditions, and from *B. mori* (Bm SGF), as well as those corresponding to RS produced by both species of silkworms (named as Scr RS and Bm RS, respectively).

### 3.4. Evaluation of Mechanical Properties

The mechanical properties of fragments (3 cm in length) of SGFs, produced from *S. c. ricini* and *B. mori*, were evaluated in the dry state in order to detect potential differences between the fibres obtained using different concentrations of acetic acid, as well as the differences with the fibres of *B. mori*, obtained by the previously optimized standard protocol. On the other hand, the same characterization was also carried out with fragments (3 cm in length) of braided scaffolds produced from SGFs of both species (manufacturing procedure explained below). In this case, the scaffolds were previously hydrated for 48 h in PBS 1×, in order to evaluate the mechanical behaviour under conditions similar to those of an in vivo environment.

Tensile tests were performed using a universal test frame machine (Qtest; MTS Systems, Eden Prairie, MN, USA). The mechanical properties of specimens were recorded with a crosshead speed of 0.1 mm·s^−1^ and a load cell of 200 N, under ambient conditions. The diameters of the SGFs, as well as the width and thickness of the braided scaffolds were determined, before each test, with an electronic digital micrometre (Mitutoyo Digimatic Micrometre 0–25 mm, resolution of 0.001 mm and an accuracy of ±2 μm). The elastic modulus (GPa or MPa), tensile strength (MPa) and strain at break (%) were determined using the stress–strain curves. The elastic modulus was calculated in the linear elastic portion of the stress–strain curves generated. Each test was performed at least three times per condition.

### 3.5. Braided of Silkworm Gut Fibres

Nine SGFs from *S. c. ricini* were manually weaved in a three-strand braid (named as Scr SGFB) with the aim to test the mechanical properties and the biocompatibility of these SGFs (Figure 7). The 2% *v*/*v* acetic acid dissolution manufacturing treatment was chosen, according to our previous studies [23,25], and three SGFs from *B. mori* silk glands were also braided for study comparisons (named as Bm SGFB). Both types of SGFs were previously hydrated in PBS 1× during 48 h, and the same tension was maintained throughout the braiding process. In the case of biocompatibility assays, the braided materials were sterilized by autoclaving (121 °C, 20 min) and stored at 4 °C until the seeding of the cells.

### 3.6. Cell-Based In Vitro Study

#### 3.6.1. Cell Culture

Human dermal fibroblasts (HDF (106-05a) cell line, ECACC N° 06090715) were chosen to test the cellular adhesion and proliferation. The viability and cell number were determined by trypan blue staining in a Neubauer chamber, and the cells were tested for the absence of mycoplasma before performing the experiments. Briefly, the cells were cultured in a DMEM/F-12 (1:1) expansion medium (supplemented with 5% foetal bovine serum (FBS), 100 U·mL^−1^ penicillin and 100 μg·mL^−1^ streptomycin) at 37 °C in a humidified atmosphere with 5% CO_2_. The medium was carefully replaced twice a week, and the cells were allowed to grow until the culture reached 80% confluence. All the chemicals used for cell culture were purchased from Sigma-Aldrich (St. Louis, MO, USA) and Gibco (Paisley, UK); Nunc (Roskilde, Denmark) provided the culture plates.

#### 3.6.2. Biocompatibility and Proliferation Assay

In order to seed the braided scaffolds, pieces of SGFBs were previously positioned into 12-well cell culture plates, fixed to the well at their ends with a drop of 3140 RTV Coating (Dow Corning, Midland, MI, USA) and incubated for two hours, at 37 °C, with 2 mL of pure FBS to facilitate the initial adhesion. Cells at 75–80% confluence were detached with 0.25% trypsin/1 mM EDTA, to proceed with the seeding of the materials after removing the excess of FBS in the wells. A volume of 3 µL containing 10,500 cells was seeded in each piece of SGFB without draining off the material. Afterwards, the plates were placed in an incubator for 30 min to allow the cells to settle, and the same seeding process was repeated before 2 mL of medium was added to each well. The medium was carefully replaced twice a week during the growing of the cells. Cell proliferation was evaluated 4 d and 10 d after seeding, using PrestoBlue (PB) reagent (Invitrogen, Thermo Fisher Scientific, Waltham, MA, USA). This is a resazurin-based membrane permeable solution which does not require cell lysis. PB quantitatively analyses the proliferation of metabolically active cells by the mitochondrial reduction of resazurin to a red fluorescent compound called resorufin. As a consequence, the reagent exhibits a change in colour, as well as a shift in its fluorescence. The SGFBs were extracted from the wells and transferred to a new plate in order to be incubated with a 10% solution of PB for 4 h at 37 °C in a 5% CO_2_ humidified atmosphere. The solution was then removed and relative fluorescence (RF) was measured using a Synergy MX microplate reader (Biotek Instruments, Winooski, VT, USA) with an excitation wavelength of 570 nm and an emission wavelength of 610 nm. The experiment was performed with 4 replicates, and SGFBs without cells were used as a control to ensure that the material does not get reduced in the assay and does not interfere with results from cells.

#### 3.6.3. Scanning Electron Microscopy

SEM was used to visualize the proliferation and morphological characteristics of the cells growing on the SGFBs 10 d after the seeding. Samples were fixed with 3% glutaraldehyde in 0.1 M cacodylate buffer for 1 h. Then, they were rinsed and post-fixed in osmium tetroxide for 1 h, before being dehydrated through increasing concentrations of ethanol (30, 50, 70, 90 vol.%), with a final dehydration in absolute alcohol. After this, they were dried by the critical-point method and gold-coated. The materials were observed using a scanning electronic microscope Jeol T-6100 at 15 kV.

### 3.7. Statistical Analysis

For the statistical analyses, the IBM SPSS Statistics software (Ver. 25) was used. As the data complied with the normality and homogeneity of variance requirements, they were compared by means of parametric tests: ANOVA followed by Bonferroni’s post hoc multiple *t*-test. In every situation, the significance level was set to *p* < 0.05.

## 4. Conclusions

This work demonstrates the feasibility of manufacturing high-performance fibres from the silk glands of *S. c. ricini*, by means of soaking them in an acetic acid dissolution and a subsequent stretching step. Some changes in the resulting fibres are detected according to the concentration of acetic acid used during its processing. The fibres obtained from *B. mori* present a larger diameter and higher degree of crystallinity (containing more β-sheet and less random coil structures) than those produced from *S. c. ricini*, which is reflected in higher values of elastic modulus and lower values of strain at break (in a dry state). Moreover, a decrease in the values of strain at break was observed in the fibres obtained from *S. c. ricini* as the concentration of acetic acid employed increased during the manufacture. The maximum average values of tensile strength and strain at break were those obtained in the SGFs produced from *S. c. ricini* using the dissolution of acetic acid at 2% *v*/*v*, also observing less roughness on its surface.

On the other hand, the study of the braided scaffolds (in the wet state) showed higher values of tensile strength and strain at break in the case of *S. c. ricini* materials and similar values of elastic modulus, compared to those of *B. mori*, with both kinds of scaffolds displaying optimal biocompatibility using a fibroblast cell line.

As far as we know, this work is the first to address the manufacture and characterization of this type of fibres using the eri silkworm, comparing them with those produced from the mulberry silkworm, and proposing the manufacture of scaffolds by braiding these fibres with a potential application in tissue engineering. However, this is only a first step, and the molecular aspects that concern the differences between the fibres produced must be investigated in-depth, as well as evaluating the behaviour of the scaffolds in future in vivo studies.

## Figures and Tables

**Table 1:** Infrared crystallinity indices of silkworm gut fibres (SGF) produced from silk glands of *S. c. ricini* (Scr SGF) under different experimental conditions and from *B. mori* (Bm SGF), as well as those correspondent to raw silk fibroins produced by both species of silkworms (named as Scr RS and Bm RS, respectively). The values are calculated as the ratio between the measured absorbance at 1625 and 1650 cm^−1^ (for the amide I peak) and between 1515 and 1538 cm^−1^ (for the amide II peak). Expressed as average values ± standard deviation.

	Infrared Crystallinity Index	
	Amide I	Amide II
**Bm SGF Ac 2%**	1.33 ± 0.09	1.12 ± 0.06
**Scr SGF Ac 2%**	1.08 ± 0.11	1.04 ± 0.04
**Scr SGF Ac 10%**	1.10 ± 0.05	1.04 ± 0.02
**Scr SGF Ac 20%**	1.13 ± 0.05	1.07 ± 0.05
**Bm RS**	0.95 ± 0.03	1.01 ± 0.01
**Scr RS**	1.20 ± 0.04	1.03 ± 0.04

**Table 2:** Assignment of the elementary contributions in the amide I region [56].

Wavenumber (cm^−1^)	Assignment
1594–1609	Tyr side chain/aggregated strand
1610–1620	Aggregate strand/intermolecular β-sheet
1621–1627	Intermolecular β-sheet
1628–1637	Intramolecular β-sheet
1638–1655	Random coil
1656–1662	α-helix
1663–1694	β-turn
1696–1703	Intermolecular β-sheet

**Table 3:** Contribution of the different secondary structures to the microstructure of silkworm gut fibres (SGF) produced from silk glands of *S. c. ricini* (Scr SGF) under different experimental conditions and from *B. mori* (Bm SGF), as well as those correspondent to raw silk fibroins produced by both species of silkworms (named as Scr RS and Bm RS, respectively). Expressed as average values ± standard deviation.

Sample	β-Sheet (%)	α-Helix (%)	Random Coil (%)
**Bm SGF Ac 2%**	59.2 ± 5.2	8.5 ± 0.7	11.3 ± 3.0
**Scr SGF Ac 2%**	49.0 ± 2.7	11.0 ± 0.9	17.2 ± 1.8
**Scr SGF Ac 10%**	49.5 ± 1.0	10.5 ± 1.3	18.1 ± 1.1
**Scr SGF Ac 20%**	49.2 ± 1.9	12.0 ± 1.2	17.9 ± 0.5
**Bm RS**	44.7 ± 1.2	11.6 ± 0.2	20.5 ± 0.9
**Scr RS**	51.9 ± 0.5	10.3 ± 0.2	17.1 ± 0.5

**Table 4:** Values of diameter and mechanical properties of silkworm gut fibres produced from silk glands of *S. c. ricini* (Scr SGF), under different experimental conditions, and from *B. mori* (Bm SGF), as described in a previous work [23]. The abbreviations include at the end the concentration of acetic acid dissolution used during its production (2%, 10% or 20% *v*/*v*). Data expressed as average values ± standard deviation.

	Tensile Strength (MPa)	Strain at Break (%)	Elastic Modulus (GPa)	Fibre Diameter (µm)
**Bm SGF Ac 2%**	306 ± 78	7.9 ± 3.3	11.6 ± 0.9	248 ± 46
**Scr SGF Ac 2%**	388 ± 12	65.0 ± 2.5	8.7 ± 1.0	133 ± 6
**Scr SGF Ac 10%**	387 ± 39	39.2 ± 3.6	8.7 ± 0.3	156 ± 3
**Scr SGF Ac 20%**	305 ± 64	28.0 ± 0.6	9.0 ± 1.5	128 ± 21

**Table 5:** Values of mechanical properties and cross-sectional areas of silkworm gut fibre braids produced from silk glands of *S. c. ricini* (Scr SGFB) and from *B. mori* (Bm SGFB), prehydrated in 1× PBS. Expressed as average values ± standard deviation.

	Tensile Strength (MPa)	Strain at Break (%)	Elastic Modulus (MPa)	Cross-Sectional Area (mm^2^)
**Bm SGFB**	35.6 ± 8.0	34.4 ± 5.7	112.9 ± 2.0	0.77 ± 0.06
**Scr SGFB**	65.0 ± 10.4	66.3 ± 3.7	119.6 ± 9.2	0.26 ± 0.02

## Data Availability

Not applicable.

## References

[1] Sun W., Gregory D.A., Tomeh M.A., Zhao X. (2021). Silk fibroin as a functional biomaterial for tissue engineering. Int. J. Mol. Sci..

[2] Omenetto F.G., Kaplan D.L. (2010). New Opportunities for an Ancient Material. Science.

[3] Nguyen T.P., Nguyen Q.V., Nguyen V., Le T., Le Q. (2019). Van Silk Fibroin-Based Biomaterials for Biomedical. Polymers.

[4] Holland C., Numata K., Rnjak-Kovacina J., Seib F.P. (2019). The Biomedical Use of Silk: Past, Present, Future. Adv. Healthc. Mater..

[5] Aznar-Cervantes S.D., Pagan A., Monteagudo Santesteban B., Cenis J.L. (2019). Effect of different cocoon stifling methods on the properties of silk fibroin biomaterials. Sci. Rep..

[6] Vepari C., Kaplan D.L. (2007). Silk as a biomaterial. Prog. Polym. Sci..

[7] Wang Y., Kim H.-J., Vunjak-Novakovic G., Kaplan D.L. (2006). Stem cell-based tissue engineering with silk biomaterials. Biomaterials.

[8] Zhang X., Reagan M.R., Kaplan D.L. (2009). Electrospun silk biomaterial scaffolds for regenerative medicine. Adv. Drug Deliv. Rev..

[9] Rockwood D.N., Preda R.C., Yücel T., Wang X., Lovett M.L., Kaplan D.L. (2011). Materials fabrication from *Bombyx mori* silk fibroin. Nat. Protoc..

[10] Nakazawa Y. (2003). Tightly winding structure of sequential model peptide for repeated helical region in *Samia cynthia ricini* silk fibroin studied with solid-state NMR. Protein Sci..

[11] Pal S., Kundu J., Talukdar S., Thomas T., Kundu S.C. (2013). An emerging functional natural silk biomaterial from the only domesticated non-mulberry silkworm samia ricini. Macromol. Biosci..

[12] Andiappan M., Kumari T., Sundaramoorthy S., Meiyazhagan G., Manoharan P., Venkataraman G. (2016). Comparison of eri and tasar silk fibroin scaffolds for biomedical applications. Prog. Biomater..

[13] Andiappan M., Sundaramoorthy S., Panda N., Meiyazhaban G., Winfred S.B., Venkataraman G., Krishna P. (2013). Electrospun eri silk fibroin scaffold coated with hydroxyapatite for bone tissue engineering applications. Prog. Biomater..

[14] Balan K.K., Sundaramoorthy S. (2019). Hydroentangled nonwoven eri silk fibroin scaffold for tissue engineering applications. J. Ind. Text..

[15] Silva S.S., Oliveira N.M., Oliveira M.B., Da Costa D.P.S., Naskar D., Mano J.F., Kundu S.C., Reis R.L. (2016). Fabrication and characterization of Eri silk fibers-based sponges for biomedical application. Acta Biomater..

[16] Mai-Ngam K., Boonkitpattarakul K., Jaipaew J., Mai-Ngam B. (2011). Evaluation of the properties of silk fibroin films from the non-mulberry silkworm *Samia cynthia ricini* for biomaterial design. J. Biomater. Sci. Polym. Ed..

[17] Andersson M., Johansson J., Rising A. (2016). Silk Spinning in Silkworms and Spiders. Int. J. Mol. Sci..

[18] Ling S., Qin Z., Li C., Huang W., Kaplan D.L., Buehler M.J. (2017). Polymorphic regenerated silk fibers assembled through bioinspired spinning. Nat. Commun..

[19] Madurga R., Gañán-Calvo A.M., Plaza G.R., Guinea G.V., Elices M., Pérez-Rigueiro J. (2017). Straining flow spinning: Production of regenerated silk fibers under a wide range of mild coagulating chemistries. Green Chem..

[20] Madurga R., Gañán-Calvo A.M., Plaza G.R., Guinea G.V., Elices M., Pérez-Rigueiro J. (2017). Production of High Performance Bioinspired Silk Fibers by Straining Flow Spinning. Biomacromolecules.

[21] Marden L. (1951). Spain’s Silkworm Gut. Natl. Geogr. Mag..

[22] Humphries A.M.C. (1949). The story of silk and silkworm gut. Postgrad. Med. J..

[23] Cenis J.L., Madurga R., Aznar-Cervantes S.D., Lozano-Pérez A.A., Marí-Buyé N., Meseguer-Olmo L., Plaza G.R., Guinea G.V., Elices M., Del Pozo F. (2015). Mechanical behaviour and formation process of silkworm silk gut. Soft Matter.

[24] Pérez-Rigueiro J., Ruiz V., Cenis J.L., Elices M., Guinea G.V. (2020). Lessons From Spider and Silkworm Silk Guts. Front. Mater..

[25] Pagán A., Aznar-Cervantes S.D., Pérez-Rigueiro J., Meseguer-Olmo L., Cenis J.L. (2019). Potential use of silkworm gut fiber braids as scaffolds for tendon and ligament tissue engineering. J. Biomed. Mater. Res. Part B Appl. Biomater..

[26] Cenis J.L., Aznar-Cervantes S.D., Lozano-Pérez A.A., Rojo M., Muñoz J., Meseguer-Olmo L., Arenas A. (2016). Silkworm gut fiber of *Bombyx mori* as an implantable and biocompatible light-diffusing fiber. Int. J. Mol. Sci..

[27] Ruiz V., Jiang P., Müller C., Jorge I., Vázquez J., Ridruejo A., Aznar-Cervantes S.D., Cenis J.L., Meseguer L., Elices M. (2019). Preparation and characterization of *Nephila clavipes* tubuliform silk gut. Soft Matter.

[28] Jiang P., Marí-Buyé N., Madurga R., Arroyo-Hernández M., Solanas C., Gañán A., Daza R., Plaza G.R., Guinea G.V., Elices M. (2014). Spider silk gut: Development and characterization of a novel strong spider silk fiber. Sci. Rep..

[29] Goh J.C.-H., Ouyang H.-W., Teoh S.-H., Chan C.K.C., Lee E.-H. (2003). Tissue-Engineering Approach to the Repair and Regeneration of Tendons and Ligaments. Tissue Eng..

[30] Vunjak-Novakovic G., Altman G., Horan R., Kaplan D.L. (2004). Tissue Engineering of Ligaments. Annu. Rev. Biomed. Eng..

[31] Calve S., Dennis R.G., Kosnik P.E., Baar K., Grosh K., Arruda E.M. (2004). Engineering of Functional Tendon. Tissue Eng..

[32] Gentleman E., Lay A.N., Dickerson D.A., Nauman E.A., Livesay G.A., Dee K.C. (2003). Mechanical characterization of collagen fibers and scaffolds for tissue engineering. Biomaterials.

[33] Caliari S.R., Ramirez M.A., Harley B.A.C. (2011). The development of collagen-GAG scaffold-membrane composites for tendon tissue engineering. Biomaterials.

[34] Fini M., Torricelli P., Giavaresi G., Rotini R., Castagna A., Giardino R. (2007). In vitro study comparing two collageneous membranes in view of their clinical application for rotator cuff tendon regeneration. J. Orthop. Res..

[35] Chen X., Qi Y.Y., Wang L.L., Yin Z., Yin G.L., Zou X.H., Ouyang H.W. (2008). Ligament regeneration using a knitted silk scaffold combined with collagen matrix. Biomaterials.

[36] Nirmalanandhan V.S., Rao M., Sacks M.S., Haridas B., Butler D.L. (2007). Effect of length of the engineered tendon construct on its structure–function relationships in culture. J. Biomech..

[37] Young R.G., Butler D.L., Weber W., Caplan A.I., Gordon S.L., Fink D.J. (1998). Use of mesenchymal stem cells in a collagen matrix for achilles tendon repair. J. Orthop. Res..

[38] Ouyang H.W., Goh J.C.H., Thambyah A., Teoh S.H., Lee E.H. (2003). Knitted Poly-lactide-co-glycolide Scaffold Loaded with Bone Marrow Stromal Cells in Repair and Regeneration of Rabbit Achilles Tendon. Tissue Eng..

[39] Sahoo S., Cho-Hong J.G., Siew-Lok T. (2007). Development of hybrid polymer scaffolds for potential applications in ligament and tendon tissue engineering. Biomed. Mater..

[40] Lim W.L., Liau L.L., Ng M.H., Chowdhury S.R., Law J.X. (2019). Current Progress in Tendon and Ligament Tissue Engineering. Tissue Eng. Regen. Med..

[41] Silva M., Ferreira F.N., Alves N.M., Paiva M.C. (2020). Biodegradable polymer nanocomposites for ligament/tendon tissue engineering. J. Nanobiotechnol..

[42] Altman G.H., Horan R.L., Lu H.H., Moreau J., Martin I., Richmond J.C., Kaplan D.L. (2002). Silk matrix for tissue engineered anterior cruciate ligaments. Biomaterials.

[43] Teuschl A., Heimel P., Nürnberger S., van Griensven M., Redl H., Nau T. (2016). A Novel Silk Fiber–Based Scaffold for Regeneration of the Anterior Cruciate Ligament. Am. J. Sports Med..

[44] Ran J., Hu Y., Le H., Chen Y., Zheng Z., Chen X., Yin Z., Yan R., Jin Z., Tang C. (2017). Ectopic tissue engineered ligament with silk collagen scaffold for ACL regeneration: A preliminary study. Acta Biomater..

[45] Fan H., Liu H., Toh S.L., Goh J.C.H. (2008). Enhanced differentiation of mesenchymal stem cells co-cultured with ligament fibroblasts on gelatin/silk fibroin hybrid scaffold. Biomaterials.

[46] Ni T., Senthil-Kumar P., Dubbin K., Aznar-Cervantes S.D., Datta N., Randolph M.A., Cenis J.L., Rutledge G.C., Kochevar I.E., Redmond R.W. (2012). A photoactivated nanofiber graft material for augmented Achilles tendon repair. Lasers Surg. Med..

[47] Li H., Fan J., Sun L., Liu X., Cheng P., Fan H. (2016). Functional regeneration of ligament-bone interface using a triphasic silk-based graft. Biomaterials.

[48] Amirikia M., Shariatzadeh S.M.A., Jorsaraei S.G.A., Mehranjani M.S. (2018). Auto-fluorescence of a silk fibroin-based scaffold and its interference with fluorophores in labeled cells. Eur. Biophys. J..

[49] Bhat N.V., Nadiger G.S. (1980). Crystallinity in silk fibers: Partial acid hydrolysis and related studies. J. Appl. Polym. Sci..

[50] Nadiger G.S., Bhat N.V. (1985). Effect of plasma treatment on the structure and allied textile properties of mulberry silk. J. Appl. Polym. Sci..

[51] Jao W.C., Yang M.C., Lin C.H., Hsu C.C. (2012). Fabrication and characterization of electrospun silk fibroin/TiO_2_ nanofibrous mats for wound dressings. Polym. Adv. Technol..

[52] Bae Y.J.S.K.I.C. (2021). Crystallinity change of silkworm variety cocoons by heat treatment. Int. J. Ind. Entomol..

[53] Ayutsede J., Gandhi M., Sukigara S., Micklus M., Chen H.E., Ko F. (2005). Regeneration of *Bombyx mori* silk by electrospinning. Part 3: Characterization of electrospun nonwoven mat. Polymer.

[54] Um I.C., Kweon H.Y., Lee K.G., Park Y.H. (2003). The role of formic acid in solution stability and crystallization of silk protein polymer. Int. J. Biol. Macromol..

[55] Kim J.H., Sheikh F.A., Ju H.W., Park H.J., Moon B.M., Lee O.J., Park C.H. (2014). 3D silk fibroin scaffold incorporating titanium dioxide (TiO_2_) nanoparticle (NPs) for tissue engineering. Int. J. Biol. Macromol..

[56] Jung C. (2000). Insight into protein structure and protein-ligand recognition by Fourier transform infrared spectroscopy. J. Mol. Recognit..

[57] Cilurzo F., Gennari C.G.M., Selmin F., Marotta L.A., Minghetti P., Montanari L. (2011). An investigation into silk fibroin conformation in composite materials intended for drug delivery. Int. J. Pharm..

[58] Guo C., Li C., Vu H.V., Hanna P., Lechtig A., Qiu Y., Mu X., Ling S., Nazarian A., Lin S.J. (2020). Thermoplastic moulding of regenerated silk. Nat. Mater..

[59] Nakazawa Y., Asakura T. (2002). Heterogeneous exchange behavior of *Samia cynthia ricini* silk fibroin during helix-coil transition studied with ^13^C NMR. FEBS Lett..

[60] Moseti K.O., Yoshioka T., Kameda T., Nakazawa Y. (2019). Aggregation state of residual α-helices and their influence on physical properties of *S. c. ricini* Native Fiber. Molecules.

[61] Maganaris C.N., Narici M.V. (2005). Mechanical properties of tendons. Tendon Injuries.

[62] Li G., Gil J., Kanamori A., Woo S.L. (1999). A validated three-dimensional computational model of a human knee joint. J. Biomech. Eng..

[63] Blankevoort L., Huiskes H.W.J. (1991). Ligament-bone interaction in a three-dimensional model of the knee. J. Biomech. Eng. Trans. ASME.

[64] Pioletti D.P., Rakotomanana L.R., Benvenuti J.F., Leyvraz P.F. (1998). Viscoelastic constitutive law in large deformations: Application to human knee ligaments and tendons. J. Biomech..

[65] Seral García B., Cegoñino Banzo J., García Aznar J.M., Doblaré Castellano M., Seral Iñigo F. (2003). Simulación en 3D con elementos finitos de un modelo de prótesis de rodilla. Rev. Esp. Cir. Ortop. Traumatol..

[66] Font Tellado S., Bonani W., Balmayor E.R., Foehr P., Motta A., Migliaresi C., Van Griensven M. (2017). Fabrication and Characterization of Biphasic Silk Fibroin Scaffolds for Tendon/Ligament-to-Bone Tissue Engineering. Tissue Eng. Part A.

[67] Sharifi-Aghdam M., Faridi-Majidi R., Derakhshan M.A., Chegeni A., Azami M. (2017). Preparation of collagen/polyurethane/knitted silk as a composite scaffold for tendon tissue engineering. Proc. Inst. Mech. Eng. H.

[68] Lujerdean C., Baci G., Cucu A. (2022). The Contribution of Silk Fibroin in Biomedical Engineering. Insects.

